# Bioinformatics-Based Analysis of the lncRNA-miRNA-mRNA Network and TF Regulatory Network to Explore the Regulation Mechanism in Spinal Cord Ischemia/Reperfusion Injury

**DOI:** 10.3389/fgene.2021.650180

**Published:** 2021-04-27

**Authors:** Dan Wang, Limei Wang, Jie Han, Zaili Zhang, Bo Fang, Fengshou Chen

**Affiliations:** Department of Anesthesiology, The First Hospital of China Medical University, Shenyang, China

**Keywords:** spinal cord ischemia/reperfusion injury, competing endogenous RNAs, network, bioinformatics analysis, lncRNA

## Abstract

**Background:**

Spinal cord ischemia/reperfusion injury (SCII) is a catastrophic complication involved with cardiovascular, spine, and thoracic surgeries and can lead to paraplegia. Nevertheless, the molecular mechanism of SCII remain ill-defined.

**Methods:**

Expression profiling (GSE138966) data were obtained from GEO database. Then, differentially expressed (DE) lncRNAs and DEmRNAs were screened out with *p* < 0.05, and | fold change| > 1.5. Aberrant miRNAs expression in SCII was obtained from PubMed. Functional enrichment analysis of overlapping DEmRNAs between predicted mRNAs in miRDB database and DEmRNAs obtained from GSE138966 was performed using cluster Profiler R package. The lncRNA-miRNA-mRNA competitive endogenous RNA (ceRNA) network was established in light of ceRNA theory. The key lncRNAs in the ceRNA network were identified by topological analysis. Subsequently, key lncRNAs related ceRNA-pathway network and transcription factors (TFs)-mRNAs network were constructed. Simultaneously, the expression levels of hub genes were measured via qRT-PCR.

**Results:**

The results in this study indicated that 76 miRNAs, 1373 lncRNAs, and 4813 mRNAs were differentially expressed in SCII. A SCII-related ceRNA network was constructed with 154 ncRNAs, 139 mRNAs, and 51 miRNAs. According topological analysis, six lncRNAs (NONRATT019236.2, NONRATT009530.2, NONRATT026999.2, TCONS_00032391, NONRATT023112.2, and NONRATT021956.2) were selected to establish the ceRNA-pathway network, and then two candidate hub lncRNAs (NONRATT009530.2 and NONRATT026999.2) were identified. Subsequently, two lncRNA-miRNA-mRNA regulatory axes were identified. NONRATT026999.2 and NONRATT009530.2 might involve SCII via miR-20b-5p/Map3k8 axis based on the complex ceRNA network. SP1 and Hnf4a acting as important TFs might regulate Map3k8. Furthermore, qRT-PCR results showed that the NONRATT009530.2, NONRATT026999.2, Map3k8, Hfn4a, and SP1 were significantly upregulated in SCII of rats, while the miR-20b-5p was downregulated.

**Conclusion:**

Our results offer a new insight to understand the ceRNA regulation mechanism in SCII and identify highlighted lncRNA-miRNA-mRNA axes and two key TFs as potential targets for prevention and treatment of SCII.

## Introduction

Spinal cord ischemia/reperfusion injury (SCII) is a catastrophic complication result from cardiovascular, spine, and thoracic surgeries and can lead to paraplegia (Wang X. et al., 2019; Fang et al., 2020). The incidence of paraplegia in patients undergoing thoracoabdominal aortic surgery is as high as 14% (Fu et al., 2020). Multiple pathological changes can result in SCII including oxidative stress, blood spinal cord barrier (BSCB), neuroinflammation, neuronal apoptosis, and autophagy, which are related with the occurrence and progression of SCII (Fang et al., 2016; Li X. Q. et al., 2016; Chen et al., 2020a,b). Therefore, inhibition of these damaging pathogeneses is widely assessed for treating spinal cord injury. Although numerous therapeutic interventions have been applied to improve the neurological function after SCII, there are still no efficacious approaches for prevention and treatment of SCII. Consequently, elaborating the molecular mechanism of SCII is important for clinical practice.

Non-coding RNA (ncRNA), including circular RNAs (circRNAs), microRNAs(miRNAs), long non-coding RNAs (lncRNAs), transfer RNAs (tRNAs), ribosomal RNAs (rRNAs), and small nuclear RNAs (snRNAs), is a class of RNA molecules that were not translated into a protein (Chandran et al., 2017). Long non-coding RNAs (lncRNAs), a class ncRNA with a non-protein-coding function > 200 nucleotides, have the ability to regulate multiple biological processes, such as inflammation, apoptosis, autophagy, and transcriptional modifications (Zhou et al., 2020a). So far, although several studies have suggested that lncRNAs acted significant roles in the pathogenesis of SCII (Liu et al., 2018; Qiao et al., 2018; Jia et al., 2019), the functions and mechanisms of most lncRNAs in SCII remained unclear. miRNAs are a class of ncRNAs of 18–25 nucleotides in length and regulate their target genes translation (Yin et al., 2020). Dysregulated miRNAs appeared and could participate in axon regeneration, as well as apoptosis and inflammatory responses through multiple pathways following SCII (Liu et al., 2020). Competitive endogenous RNA (ceRNA) hypothesis was mentioned by Salmena et al. (2011). According to this hypothesis, any RNA transcript with miRNA response elements (MREs) could be combined with miRNA to regulate the expression of RNAs with the same MREs (Jiang et al., 2020). There is growing evidence that lncRNA, mRNA and other RNAs could compete with miRNAs by acting as miRNA sponges via sharing at least one miRNA response elements (MREs) (Long et al., 2019; Zhou et al., 2019). Based on ceRNA,lncRNAs and mRNAs may have the same MREs. Hence, the inhibitory effect of miRNA on mRNA might be canceled when miRNAs is combined with MRE on lncRNA. Recently, accumulating studies have demonstrated that the ceRNA network has become increasingly important in the occurrence and progression of diverse diseases, including cancers, Parkinson’s disease, myocardial ischemia/reperfusion injury, and cerebral infarction (Zou et al., 2019; Qi et al., 2020; Zhang R. et al., 2020; Zhang X. et al., 2020). Nonetheless, few studies of ceRNA network in SCII have been reported (Liu et al., 2018; Qiao et al., 2018). Transcription factors (TFs) are involved in regulating post-transcription and transcription of genes via binding to particular DNA sequences (Ye et al., 2020). However, the functions and interrelationships of lncRNAs, miRNAs, mRNAs, and TFs in SCII are not well clarified.

**FIGURE 1:**
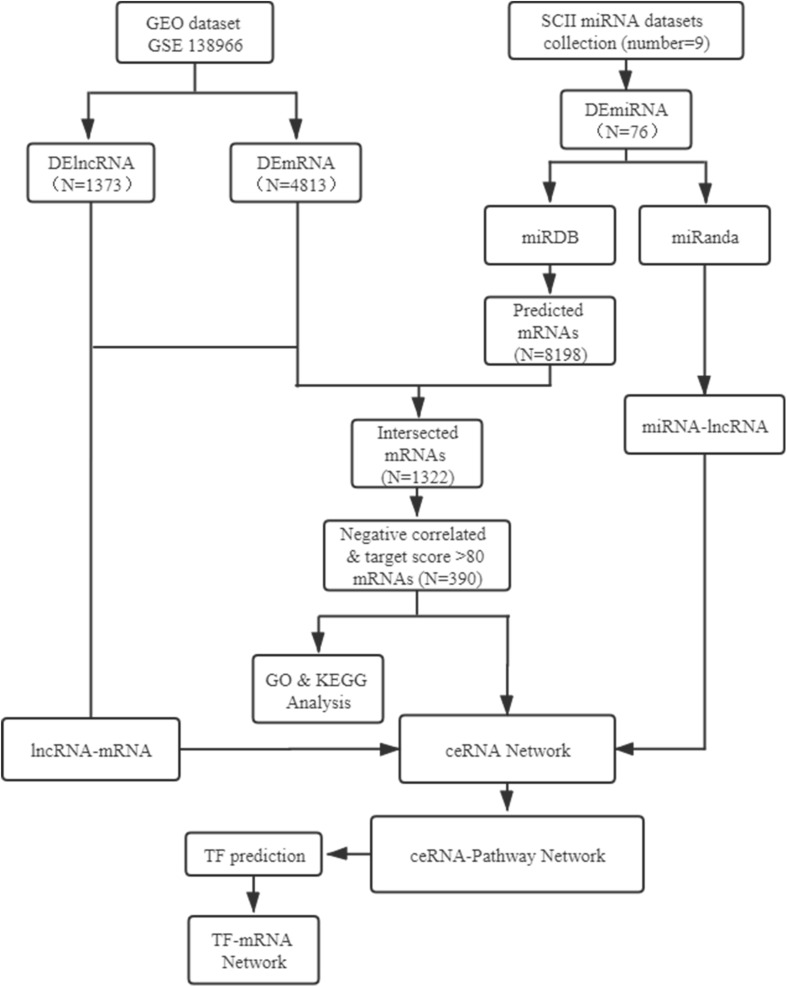
Flow-chat of lncRNA-miRNA-mRNA ceRNA network and TF regulatory network analysis. SCII, spinal cord ischemia/reperfusion injury; DElncRNAs, differentially expressed lncRNAs; DEmiRNAs, differentially expressed miRNAs; DEmRNAs, differentially expressed mRNAs; TF, transcription factor.

In the present study, the expression profiles of TFs, miRNAs, mRNAs and lncRNAs were systematic analyzed based on GSE138966 from NCBI GEO website and miRNA data from PubMed. The lncRNA-miRNA-mRNA ceRNA regulatory network and TFs analysis of ceRNA-Pathway related mRNAs network was further constructed to establish key lncRNA-miRNA-mRNA axes and TFs in SCII. The flow-chat of the present study was shown in Figure 1.

## Materials and Methods

### Data Collection

The data were obtained from the GEO database and PubMed. The mRNA and lncRNA expression profiles were obtained from dataset GSE138966 (3 pairs of rat post 48 h of SCII and sham tissues). About miRNA data, we used keywords “miRNA” and “Spinal Cord Ischemia Reperfusion Injury” to cast about for the existing SCII miRNA expression post 48 h of SCII on PubMed. The species in the selected studies were limited to rats.

### Identification of Differentially Expressed lncRNAs (DElncRNAs), DEmiRNAs, and DEmRNAs

Two-class differential analysis was applied to explore DEmRNAs and DElncRNAs between SCII and sham rats. The cut off criterion of dysregulated mRNAs and lncRNAs was a | fold change| > 1.5. R package (Robinson et al., 2010), a public available statistical computing software, was used to performed Student’s *t*-test (Mak et al., 2020). *p* < 0.05 was identified statistically significant. The information of the SCII and control miRNA sequencing samples, DEmiRNAs detection methods used in the articles were obtain by reading these studies carefully. The details of up and downregulation DEmiRNAs in SCII by compared with the sham samples were extracted.

### Prediction of Target Genes and lncRNAs for DEmiRNAs

Accurate prediction of miRNA targets is the key to characterizing miRNA functions. miRDB, consisting of 1.5 billion reads from 52 RNA samples, represents the largest of its kind for miRNA target analysis (Chen and Wang, 2020). In this study, DEmiRNA-mRNA interactions was predicted by using miRDB^1^ (Wong and Wang, 2015). Nothing but target genes score more than 80 were considered as mRNAs that interacted with DEmiRNAs. The final DEmRNAs were obtained from overlapping predicted mRNAs in miRDB database and DEmRNAs from GSE138966. miRanda^2^ was used to predict the target lncRNAs of the DEmiRNAs. The correlation between lncRNA and mRNA expression was calculated via Pearson’s correlation coefficient. The pairs of lncRNA-mRNA with score > 0.99 and *p* < 0.05 were considered as target pairs (Tao et al., 2020).

### Gene Ontology (GO) Enrichment Analysis and Kyoto Encyclopedia of Genes and Genomes (KEGG) Pathway Analysis

For the purpose of exploring the functions of the final DEmRNAs, Gene ontology and KEGG pathway analysis of final DEmRNAs were performed using the cluster Profiler R package [5]. We set *p* < 0.05 and Benjamini-Hochberg corrected *p* < 0.05 as the threshold (Yin et al., 2020).

### Establishment of a lncRNA-miRNA-mRNA ceRNA Network and Topological Analysis

If the lncRNA and mRNA were both targeted and were sharing a common miRNA in a co-dysregulated lncRNA-mRNA pair, the lncRNA-miRNA-mRNA ceRNA network was determined to a co-expression competing triplet (Tao et al., 2020). To give a deeper comprehension of the action of lncRNAs in the ceRNA network, all the potential co-dysregulated competing triples were set up to construct the lncRNA-miRNA-mRNA network and visualized using the Cytoscape software (Yin et al., 2020).

To explore critical clues in complex network sets, we applied topological analysis. We calculated the betweenness centrality (BC) and degree of each node. Furthermore, the node with a degree > 5 and a larger BC value were determined to be the critical nodes in the regulatory network (Han et al., 2004).

### Constructing a Key ceRNA-Pathway Network

Cytoscape software was used to analyze the association among the candidate lncRNA, the miRNA, mRNA and the mRNA related pathways for creating the lncRNA-miRNA-mRNA-Pathway network. The nodes of different shapes and colors represented different lncRNAs, miRNAs, mRNAs and pathways in the network. The crucial lncRNAs were identified through the lncRNA-miRNA-mRNA-Pathway network.

### Prediction of TFs for ceRNA-Pathway Related mRNAs

GTRD^3^ was used to predict the possible binding sites of TFs in a certain region near the gene location (1000 upstream, 100 downstream). SiteCount was used to judge the possibility of the predicted results. The higher the value was, the more binding sites the TF predicted.

### Rat SCII Model

Two hundred to two hundred and fifty gram male Sprague-Dawley (SD) rats were obtained from the Animal Center of China Medical University. The Ethics Committee of China Medical University approved the present study. All animals (*n* = 6) were housed for at least 1 week before the surgical operation. According to previously reports, a cross-clamped aortic arch was maintained for 14 min to establish SCII model. In a nutshell, intraperitoneal injection of 4% pentobarbital sodium (50 mg/kg; Beyotime Biotechnology, China) was followed by mechanical ventilation after endotracheal intubation (tidal 15 mL/kg, breathing frequency 80–100 times/min, respiratory rate 1:1). Body temperatures were maintained at 37.5 ± 0.5°C with monitoring. Then, the muscle tissue was separated layer by layer in the right decubitus position, and the aortic arch was cross-clamped by non-invasive artery clamp for 14 min between the left carotid artery and the left subclavian artery to induced ischemia under direct vision. The same procedure without any block was performed on sham-operated rats. After the operation, the rats were placed in clean cages to keep warm and prevent infection (Chen et al., 2020a,b).

### Quantitative Reverse Transcription-Polymerase Chain Reaction (qRT-PCR)

Segments L4-L6 of the spinal cord at 48 h after SCII were collected to extract total RNA with using Trizol reagent (Takara, otsu, Japan). Then we used Prime-Script RT reagent Kit with gDNA Eraser (Takara) to reverse-transcribe RNA into cDNA using Prime-Script RT reagent Kit with gDNA Eraser (Takara). The mRNA, lncRNA and TF expression levels were determined using the SYBR PremixEx Taq II kit (Takara) with GAPDH as an internal control on Applied Biosystems 7500 real Time PCR system. The miRNA expression levels were determined using SYBR Premix qRT-PCR (Takara) on Applied Biosystems 7500 Real Time PCR system with U6 as an internal control (Santos et al., 2020). Table 1 demonstrated the primer sequences used in the present study. We calculated data through the 2^–ΔΔCt^ method.

**TABLE 1:** qRT-PCR primers sequences used in this study.

**miRNA/gene**	**Forward primer**	**Reverse primer**
miR-20b-5p	5′-CAAAGUGCTCATAGTGCAGGTAG-3′	−
NONRATT009530.2	5′-GTGCTCTTGTCTGTGTCTGTGTCC-3′	5′-CACTTCTGAAGCCACTGCCACTC-3′
NONRATT026999.2	5′-GTGAAGGTGCTTAACGCCAGGAG-3′	5′-GCCGCAGAACACGCATCCTC-3′
Map3k8	5′-CGTGAGTAGTGGTGTCTGCCTTG-3′	5′-GGAATCTGTGTCTGCTGCTGAGTG-3′
SP1	5′-CTGCAAGGGTCTGATTCTCTA-3′	5′-AGCTTGTCCACCTTGAACTA-3′
Hnf4a	5′-TGCCAACCTCAACTCATCCAACAG-3′	5′-TCCTCACGCTCCTCCTGAAGAATC-3′
GAPDH	5′-GGGAAACTGTGGCGTGAT-3′	5′-GGGTGTCGCTGTTGAAGT-3′
U6	5′-CTCGCTTCGGCAGCACA-3′	5′-AACGCTTCACGAATTTGCGT-3′

### Statistical Analysis

SPSS 25.0 (SPSS, United States) was utilized for data analysis. All results were recorded as mean ± standard deviation. To compare the differences of qRT-PCR results, two-tailed Student’s *t*-test was used, *p* < 0.05 was identified statistically significant. Pearson’s correlation was used to calculate the correlation between DElncRNAs and DEmRNAs.

## Results

### Identification of DEmiRNAs, DElncRNAs, and DEmRNAs

PubMed was used to scan the DEmiRNAs. In the existing SCII miRNA expression profiling in rats, DEmiRNAs in the spinal cord tissues post 48 h of SCII rats and compared with sham rats were provided in 9 datasets (Table 2). SCII miRNA datasets were named based on their author acronyms and year of publication for further study. As a result, 78 DEmiRNAs were identified in 9 SCII miRNA expression datasets. In these DEmiRNAs, miR-320a were not clearly found in the miRDB datasets. In addition, it was noted that miR-22-3p showed inconsistent expression trend in three different datasets: LJA2016, ZGL2020, and HF2020, and miR-632 also showed conversed expression trend in two different datasets: ZGL2020 and LXQ2016. Besides, we found that miR-323 has two mature miRNAs (miR-323-5p and miR-323-3p). Therefore, a total of 76 DEmiRNAs (Table 3) (35 upregulated and 41 downregulated) were selected for subsequently analysis.

**TABLE 2:** The basic characteristics of SCII DEmiRNA datasets.

**References**	**Data set**	**species**	**Samples**	**Assay/sequencing type**	**Validated**
Hu et al., 2013	HJR2013	Rat	Spinal cord tissues	microRNA microarrays	qRT-PCR
Li et al., 2015	LL2015	Rat	Spinal cord tissues	qRT-PCR	−
Li J. A. et al., 2016	LJA2016	Rat	Spinal cord tissues	microarray analysis	−
Li X. Q. et al., 2016	LXQ2016	Rat	Spinal cord tissues	microRNA microarrays	qRT-PCR
Li et al., 2018	LXQ2018	Rat	Spinal cord tissues	qRT-PCR	−
Liu et al., 2018	LY2018	Rat	Spinal cord tissues	RT-PCR	−
Wang X. et al., 2019	WXY2019	Rat	Spinal cord tissues	qRT-PCR	−
Fang et al., 2020	HF2020	Rat	Spinal cord tissues	qRT-PCR	−
Liu et al., 2020	ZGL2020	Rat	Spinal cord tissues	microRNA microarrays	qRT-PCR

**TABLE 3:** The total of 76 DEmiRNAs.

**Upregulated miRNAs**	**Downregulated miRNAs**
rno-miR-204-3p	rno-miR-125b-5p
rno-miR-365-3p	rno-miR-199a-3p
rno-miR-672-3p	rno-miR-210-3p
rno-miR-760-5p	rno-miR-146a-5p
rno-miR-369-5p	rno-miR-493-5p
rno-miR-133a-5p	rno-miR-181b-5p
rno-miR-505-5p	rno-miR-21-5p
rno-miR-466d	rno-miR-381-3p
rno-miR-132-5p	rno-miR-99a-3p
rno-miR-665	rno-miR-1298
rno-miR-463-3p	rno-miR-487b-3p
rno-miR-183-3p	rno-miR-291a-3p
rno-miR-34c-3p	rno-miR-377-5p
rno-miR-200b-3p	rno-miR-144-5p
rno-miR-466c-5p	rno-miR-201-5p
rno-miR-465-3p	rno-miR-129-2-3p
rno-miR-185-3p	rno-miR-129-1-3p
rno-miR-105	rno-miR-20b-5p
rno-miR-155-3p	rno-miR-130b-3p
rno-miR-935	rno-miR-743a-3p
rno-miR-140-5p	rno-miR-497-5p
rno-miR-346	rno-miR-336-5p
rno-miR-873-5p	rno-miR-148a-3p
rno-miR-339-5p	rno-let-7f-5p
rno-miR-149-5p	rno-miR-125b-2-3p
rno-miR-376a-5p	rno-miR-28-5p
rno-miR-330-5p	rno-miR-375-5p
rno-miR-127-3p	rno-miR-352
rno-miR-675-5p	rno-miR-19a-3p
rno-miR-136-5p	rno-miR-1188-3p
rno-miR-30c-5p	rno-miR-1306-5p
rno-miR-323-3p	rno-miR-182
rno-miR-323-5p	rno-miR-214-3p
rno-miR-186-3p	rno-miR-1839-5p
rno-miR-376b-5p	rno-miR-499-5p
	rno-miR-3547
	rno-miR-19b-3p
	rno-miR-98-5p
	rno-miR-154-3p
	rno-miR-743b-3p
	rno-miR-542-5p

**FIGURE 2:**
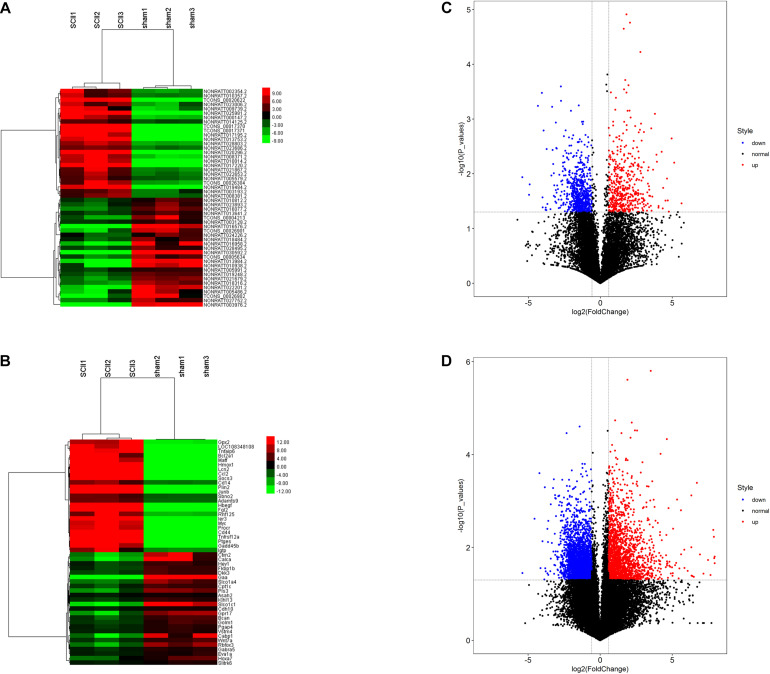
DElncRNAs and DEmRNAs in SCII and sham group. Heatmap and hierarchical clustering of the top 25 upregulated and down regulated DElncRNAs **(A)** and DEmRNAs **(B)**. Red indicates that the differentially expressed lncRNAs or DEmRNAs has a high expression value and green indicates that a differentially expressed gene has a low expression value. Volcano plot of 1373 DElncRNAs **(C)** and 4813 DEmRNAs **(D)** in SCII. The red and blue points symbolize the upregulated and downregulated DElncRNAs and DEmRNAs with statistical significance (| FC| > 1.5, *p* < 0.05).

Analysis of the GSE138966 datasets, we confirmed 1373 DElncRNAs (562 upregulated lncRNAs and 811 downregulated lncRNAs) (Supplementary Table 1) and 4813 DEmRNAs (1689 upregulated mRNAs and 3124 downregulated mRNAs) (Supplementary Table 2) in the light of the predetermined cut off values of | FC| > 1.5 and *p* < 0.05. Subsequently, hierarchical clustering analysis displayed that the expression patterns of top 25 upregulated and top 25 downregulated DElncRNAs (Figure 2A) and top 25 upregulated and top 25 downregulated DEmRNAs (Figure 2B). The volcano plots of DElncRNAs and DEmRNAs were constructed, respectively (Figures 2C,D). All of these DEmRNAs and DElncRNAs may involve in the pathological processes of SCII.

### Prediction of Target Genes and lncRNAs for DEmiRNAs

Based on miRanda, we analyzed the interactions between the DEmiRNAs and the DElncRNAs, and 6918 interactions between the DEmiRNAs and the DElncRNAs were predicted, which included 1311 lncRNAs and 76 miRNAs (Supplementary Table 3). Subsequently, the miRNAs-mRNAs target regulation network was further explored using miRDB, and we obtained 498 interactions between DEmiRNAs and DEmRNAs, which included 66 DEmiRNAs and 391 DEmRNAs (Supplementary Table 4).

### Functional Enrichment Analysis of the miRNA Targeted DEmRNAs

**FIGURE 3:**
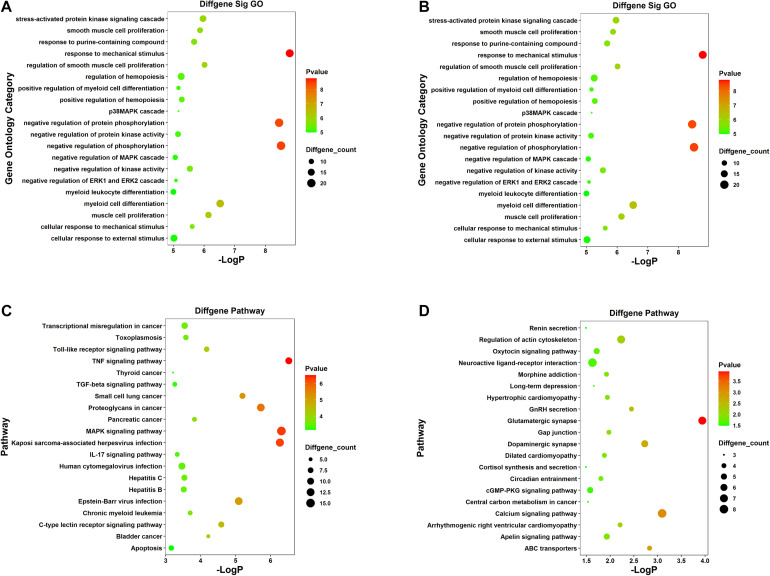
GO and KEGG pathway functional enrichment analysis of the DEmRNAs. **(A)** The top 20 most significant GO terms of upregulated mRNAs. **(B)** The top 20 most significant GO terms of downregulated mRNAs. **(C)** The top 20 most significant KEGG pathway terms of upregulated mRNAs. **(D)** The top 20 most significant KEGG pathway terms of downregulated mRNAs.

For the sake of deeper understanding of the DEmRNAs’ function, GO enrichment analysis and KEGG pathway analysis on the upregulated and downregulated genes were performed to identify potential candidate pathways or biological processes related to SCII (Supplementary Table 5). The GO analysis displayed the top 20 enriched GO terms of the upregulated and down regulated mRNAs (Figures 3A,B). In the upregulated mRNAs, the terms of biological process (BP) were enriched in “stress-activated protein kinase signaling cascade,” “negative regulation of protein phosphorylation,” and “p38MAPK cascade.” “Negative regulation of ERK1 and ERK2 cascade” and “negative regulation of MAPK cascade” also required our attention; in the downregulated mRNAs, SCII was significantly enriched in “positive regulation of neuron projection development,” “regulation of axonogenesis,” “regulation of cell morphogenesis involved in differentiation,” “axon development,” and “negative regulation of neurogenesis”; these results indicated significantly roles for these biological activities in SCII. The KEGG pathway analysis showed that upregulated mRNAs were also significantly in “Toll-like receptor signaling pathway,” “MAPK signaling pathway,” and “TNF signaling pathway,” in addition, the “IL-17 signaling pathway,” “C-type lectin receptor signaling pathway,” and “TGF-beat signaling pathway” were remarkably related to SCII (Figure 3C); then, downregulated mRNAs were enriched in “Glutamatergic synapse,” “GnRH secretion,” “Oxtocin signaling pathway,” “Neuroactive ligand-receptor interaction,” “Calcium signaling pathway” and “cGMP-PKG signaling pathway” (Figure 3D). All in all, these results implied that these DEmRNAs may act functional roles in SCII. Thus, we speculated that the miRNAs and lncRNAs, that related with these mRNAs, might participate in the similar functional processes.

### Establishment of lncRNA-miRNA-mRNA Network

**FIGURE 4:**
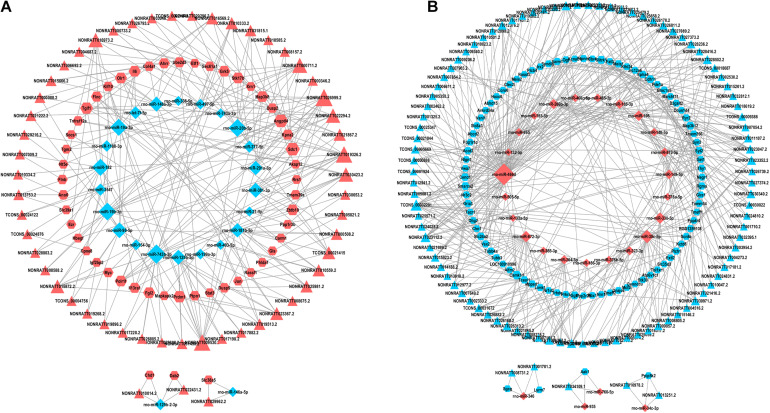
lncRNAs-miRNAs-mRNAs regulatory network. Every node symbolizes one gene, and each edge indicates the interaction between genes. The shape of triangle represents lncRNAs, diamond represents miRNAs, and regular hexagon represents mRNAs. The blue color genes symbolize downregulated lncRNAs, mRNAs and miRNAs, while the red color indicates upregulated genes. **(A)** The upregulated lncRNAs and related network. **(B)** The downregulated lncRNAs and related network.

The aforementioned interactions between lncRNA-miRNA, miRNA-mRNA, and lncRNA-mRNA were used to establish a lncRNA-miRNA-mRNA network. We built the ceRNA network after selecting the pre-treated data with *p* < 0.05 and |FC| > 2 according to the criteria of ceRNA interactions (Figure 4). 154 lncRNA nodes, 139 mRNA nodes, and 51 miRNA nodes (Supplementary Table 6) were included in the network.

### Topological Analysis of the ceRNA Network

For the purpose of identifying the hub genes in the ceRNA network that are related to SCII. The topological features of this ceRNA network were analyzed by a built-in Network Analyzer tool in Cytoscape software, including closeness centrality, degree, and betweenness (Zhou et al., 2020a). The nodes with a degree greater than 5 were identified as hub nodes according to the previous study (Han et al., 2004). 69 nodes could be determined as hub nodes including 13 mRNAs, 26 miRNAs, and 30 lncRNAs (Table 4). In addition, higher BC values of the nodes indicated an increased significance of these nodes in the regulatory network (Song et al., 2016). From the central network, we found three upregulated DElncRNAs (NONRATT019236, NONRATT009530.2, NONRATT026999.2) and three downregulated DElncRNAs (TCONS-00032391, NONRATT023112.2, NONRATT021956.2) had higher degree along with higher BC values, indicating that they may be key regulators in the SCII related ceRNA network. In Table 5, detailed information of the FC of these six alternative lncRNAs were provided.

**TABLE 4:** The list of differentially expressed genes in the lncRNAs-miRNAs-mRNAs regulatory ceRNA network (node degree > 5).

**Name**	**Betweenness centrality**	**Closeness centrality**	**Degree**	**Style**	**Type**
rno-miR-466d	0.51218231	0.45475113	67	Up	miRNA
rno-miR-30c-5p	0.19202477	0.35828877	28	Up	miRNA
TCONS_00032291	0.13638041	0.35263158	19	Down	lncRNA
rno-miR-330-5p	0.0980652	0.35078534	19	Up	miRNA
rno-miR-672-3p	0.09227397	0.3972332	19	Up	miRNA
rno-miR-105	0.0628762	0.2942899	16	Up	miRNA
rno-miR-743b-3p	0.18723038	0.35608309	15	Down	miRNA
rno-miR-200b-3p	0.06427476	0.33059211	14	Up	miRNA
rno-miR-19b-3p	0.15041392	0.36144578	13	Down	miRNA
rno-miR-20b-5p	0.1312424	0.34883721	13	Down	miRNA
rno-miR-19a-3p	0.1326247	0.31007752	12	Down	miRNA
rno-miR-182	0.12489841	0.33057851	12	Down	miRNA
NONRATT026999.2	0.09955192	0.2942899	12	Up	lncRNA
NONRATT023112.2	0.05022828	0.33895447	12	Down	lncRNA
rno-miR-185-3p	0.04457109	0.35502959	12	Up	miRNA
rno-miR-149-5p	0.0367307	0.32576985	12	Up	miRNA
NONRATT019326.2	0.21525703	0.35928144	11	Up	lncRNA
NONRATT009530.2	0.19168702	0.36474164	11	Up	lncRNA
Camk1d	0.07605598	0.40361446	11	Down	mRNA
rno-miR-98-5p	0.09080408	0.2919708	10	Down	miRNA
rno-miR-873-5p	0.03179831	0.27762431	10	Up	miRNA
rno-miR-381-3p	0.09927927	0.30781011	9	Down	miRNA
NONRATT000711.2	0.09817817	0.28103044	9	Up	lncRNA
rno-miR-125b-5p	0.0752682	0.34582133	9	Down	miRNA
rno-miR-148a-3p	0.05789145	0.30075188	9	Down	miRNA
NONRATT021956.2	0.05212423	0.30044843	9	Down	lncRNA
rno-miR-323-3p	0.03536281	0.32967033	9	Up	miRNA
TCONS_00019774	0.03441627	0.29777778	9	Down	lncRNA
NONRATT023352.2	0.02355001	0.29558824	9	Down	lncRNA
rno-miR-204-3p	0.01989314	0.35765125	9	Up	miRNA
Slitrk1	0.01385154	0.30454545	9	Down	mRNA
NONRATT022294.2	0.09576549	0.3	8	Up	lncRNA
Angptl4	0.09104243	0.2484472	8	Up	mRNA
NONRATT028502.2	0.08986369	0.29411765	8	Down	lncRNA
NONRATT016973.2	0.08342507	0.27272727	8	Up	lncRNA
rno-miR-181b-5p	0.07952858	0.32608696	8	Down	miRNA
NONRATT030423.2	0.05755366	0.32345013	8	Up	lncRNA
Sdc1	0.03704544	0.3125	8	Up	mRNA
rno-miR-291a-3p	0.02976731	0.28920863	8	Down	miRNA
NONRATT030971.2	0.02028265	0.29646018	8	Down	lncRNA
Ggt7	0.01764119	0.35387324	8	Down	mRNA
Map3k12	0.01396893	0.29955291	8	Down	mRNA
NONRATT012376.2	0.07174532	0.32697548	7	Down	lncRNA
NONRATT015872.2	0.06823347	0.318542	7	Up	lncRNA
TCONS_00005669	0.0267793	0.30781011	7	Down	lncRNA
NONRATT030340.2	0.02432357	0.26979866	7	Down	lncRNA
NONRATT023047.2	0.01498462	0.38803089	7	Down	lncRNA
NONRATT026313.2	0.01112549	0.3236715	7	Down	lncRNA
Cdh11	0.00978699	0.36086176	7	Down	mRNA
Smarca2	0.00489491	0.3236715	7	Down	mRNA
Slc24a2	0.00114177	0.33555927	7	Down	mRNA
Cend1	0.00095987	0.32057416	7	Down	mRNA
TCONS_00021415	0.09286285	0.28301887	6	Up	lncRNA
Prdm1	0.05749742	0.31114551	6	Up	mRNA
NONRATT023367.2	0.03062845	0.26728723	6	Up	lncRNA
rno-miR-665	0.03050819	0.28389831	6	Up	miRNA
rno-let-7f-5p	0.02487038	0.3125	6	Down	miRNA
NONRATT028236.2	0.02255182	0.302267	6	Down	lncRNA
NONRATT015923.2	0.02175692	0.26966292	6	Down	lncRNA
NONRATT027089.2	0.02005236	0.31662269	6	Down	lncRNA
NONRATT009857.2	0.01741403	0.30362538	6	Down	lncRNA
Tgif1	0.0162247	0.28309859	6	Up	mRNA
rno-miR-465-3p	0.0158817	0.28470255	6	Up	miRNA
NONRATT012977.2	0.01428249	0.35201401	6	Down	lncRNA
NONRATT006267.2	0.0135891	0.35387324	6	Down	lncRNA
NONRATT011107.2	0.01284285	0.3295082	6	Down	lncRNA
NONRATT023553.2	0.01258839	0.32524272	6	Down	lncRNA
rno-miR-140-5p	0.01137994	0.34010152	6	Up	miRNA
Syt2	0.00482999	0.27346939	6	Down	mRNA

**TABLE 5:** Basic information of the candidate 6 DElncRNAs.

**DE-lncRNAs**	**Fold change**	***P*-value**	**Degree**	**Betweeness centrality**	**Closeness centrality**	**Down/Up**
NONRATT026999.2	2.62678022728	0.018017	12	0.099551912	0.2942899	Up
NONRATT019326.2	2.56469830552	0.034967	11	0.21525703	0.35928144	Up
NONRATT009530.2	355.506999015	0.036493	11	0.19168702	0.36474164	Up
TCONS_00032291	−3.07336006	0.034165	19	0.13638041	0.35263158	Down
NONRATT023112.2	−3.29641383	0.033856	12	0.05022828	0.33895447	Down
NONRATT021956.2	−2.58208307	0.045792	9	0.05212423	0.30044843	Down

### Construction of ceRNA-Pathway Network

**FIGURE 5:**
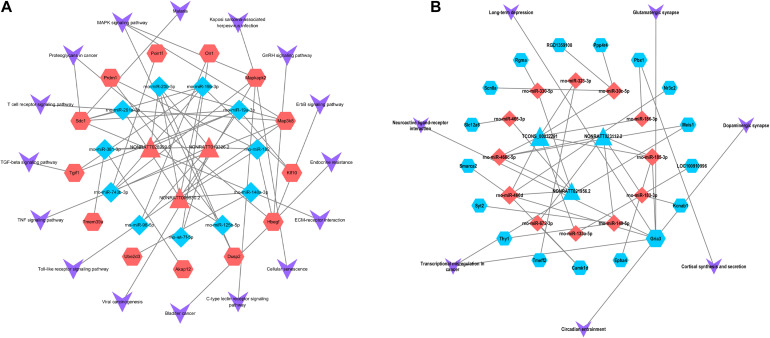
Pathway analysis of the key lncRNAs associate ceRNA network. The ceRNA-pathway network was constructed with the six candidate lncRNAs. Triangle nodes indicate dysregulated lncRNAs, regular hexagon nodes represent mRNAs, and diamond nodes represent dysregulated miRNAs. The blue nodes of lncRNAs, mRNAs and miRNAs indicate downregulated expression, while the red nodes indicate upregulated expression. The shape of V indicates the pathways in which lncRNAs, miRNAs and mRNAs might involve. **(A)** The upregulated key lncRNAs and related ceRNA-pathway network. **(B)** The downregulated key lncRNAs and related ceRNA-pathway network.

We constructed a ceRNA-pathway network to probe the signaling pathways in which the six hub lncRNAs and relative miRNAs are involved. In the network, we found that one lncRNA or one miRNA connected with different mRNAs, while one mRNA connected with several miRNAs and lncRNAs. It is interesting that different mRNAs might involve in the uniform signaling pathways. A case in point, Dusp2 expression was positively correlated with NONRATT019326.2, and it functioned via MAPK signaling pathway, while Map3k8 and Mapkap2 expression, which correlated with NONRATT009530.2 and NONRATT026999.2, also functioned with MAPK signaling pathway (Figure 5). In the network, we found that Map3k8, which was correlated with NONRATT009530.2 and NONRATT026999.2, involved in multiple signaling pathway such as: “T cell receptor signaling pathway,” “Toll-like receptor signaling pathway,” “TNF signaling pathway,” and “MAPK signaling pathway.” Additionally, NONRATTT009530.2 and NONRATT026999.2 participated in a total of 7 and 13 signaling pathways, respectively. These results indicated that NONRATT026999.2 and NONRATT009530.2 might involve in the key signaling pathways in SCII. Moreover, we exacted two lncRNA-miRNA-mRNA regulatory axes from above analysis: NONRATT026999.2/rno-miR-20b-5p/Map3k8 and NONRATT009530.2/rno-miR-20b-5p/Map3k8.

### TFs Analysis of ceRNA-Pathway Related mRNAs in SCII

**FIGURE 6:**
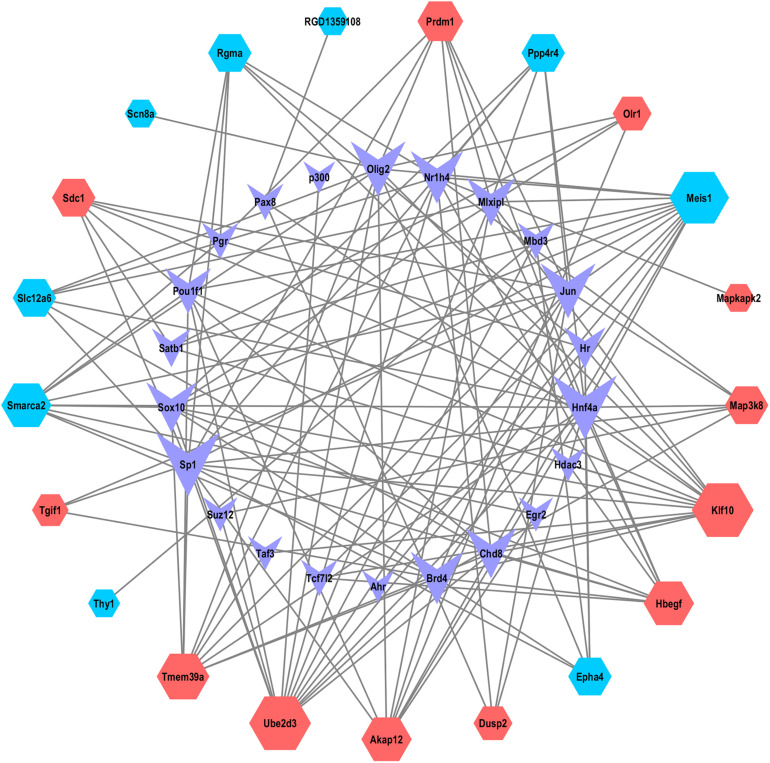
The network of TFs and ceRNA-Pathway network related mRNAs. Nodes symbolize mRNAs indicated with diamond shapes, red represents upregulated genes and blue represent downregulated genes. The shape of purple V represents TFs.

The TFs correlated with the mRNAs of the ceRNA-Pathway were further explored. As shown in the Figure 6, 43 nodes and 136 edges, and multiple ceRNA-Pathway related mRNAs connected with TFs in the transcription-regulated network (Supplementary Table 7). And we recognized that TFs, such as hepatocyte nuclear factor 4-alpha (Hnf4a), specificity protein 1(SP1), oligodendrocyte transcription factor 2 (Olig2) and transcription factor AP-1 (Jun) had a significant regulatory effect on ceRNA-related mRNAs. Interestingly, we realized that Map3k8 could be regulated by SP1 and Hnf4a.

### Dysregulated Expression of Key lncRNA-miRNA-mRNA Axes and Key TFs in Rats With SCII

**FIGURE 7:**
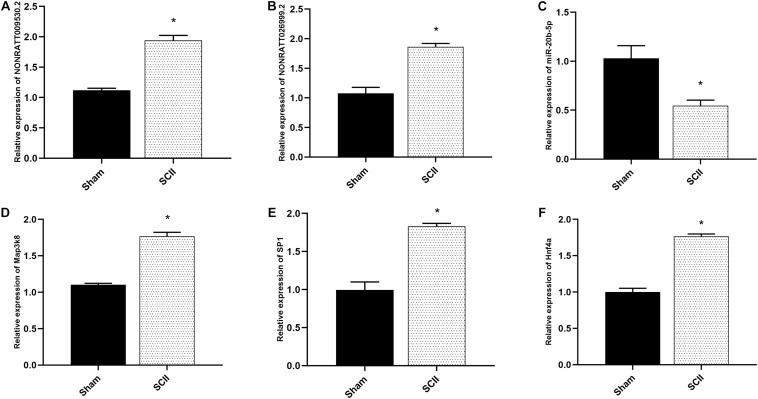
The mRNA expression of hub genes in SCII of rats. **(A,B)** The key lncRNAs expression of NONRATT009530.2 and NONRATT026999.2 in spinal cord of rats were measured by qRT-PCR post SCII, and SCII induced upregulation of NONRATT009530.2 and NONRATT026999.2 (*n* = 3). **(C)** The expression level of miR-20b-5p in spinal cord of rats in SCII (*n* = 3). **(D)** Quantification of the Map3k8 expression by qRT-PCR (*n* = 3). **(E,F)** Expression levels of SP1 and Hnf4a in a rat model of SCII (*n* = 3). Data are expressed as the mean ± standard deviation. * *p* < 0.05 vs. the sham group.

qRT-PCR was applied to measure the expression of NONRATT009530.2, NONRATT026999.2, miR-20b-5p, and Map3k8 in SCII rats and the control rats. The expression of NONRATT009530.2, NONRATT026999.2, and Map3k8 were significantly increased in SCII (*p* < 0.05), while the miR-20b-5p expression levels was markedly decreased (*p* < 0.05), as shown in Figures 7A–D. The expression levels of SP1 and Hnf4a were also increased in SCII (Figures 7E,F).

## Discussion

In recent decades, there is growing functional exploration of ncRNA, enhancing our comprehension of many biological processes. LncRNAs are regarded as a class of transcript greater than 200 nucleotides, with no coding function (Roberts et al., 2014). Accumulating studies indicated that dysregulation of lncRNAs was involved in CNS diseases (Wu et al., 2013; Riva et al., 2016; Wang Q. et al., 2020). Moreover, the expression profiles of lncRNA were related with brain development and functional diversity and lead to a variety of neurological disorders such as CNS damages (Qureshi and Mehler, 2012). With regard to this, emphasizing the possible functional acts of lncRNAs in SCII deserves due attention. In this study, we mainly explored the role of lncRNA and target genes on pathogenesis of SCII.

Previous studies have demonstrated that miRNAs and lncRNAs are potentially new regulators in prevention and treatment of SCII and their roles in regulating target genes might be crucial in the pathological mechanism of SCII (Hu et al., 2013; Li J. A. et al., 2016; Zhou et al., 2020c). In rat models of SCII, miR-125b mimic was found to protect against SCII via reducing aberrant p53 network activation-induced apoptosis and neuroinflammation through the downregulation of TP53INP1 (Li et al., 2018). miR-22-3p alleviated SCII by modulating M2 macrophage polarization via IRF5 (Fang et al., 2020). Hydrogen sulfide protected against SCII and induced autophagy via miR-30c (Li et al., 2015). TUG1 knockdown has been found to inhibit neuroinflammation of the TLR4/NF-κB/IL-1β signaling pathway after ischemia/reperfusion (I/R) by suppressing TRIL expression in SCII (Jia et al., 2019). MALAT1 overexpression was reported to induce anti-apoptosis and knockdown of MALAT1 induced pro-apoptosis in a rat model of SCII (Qiao et al., 2018). The knockdown of CasC7 could promote cell apoptosis in SCII via increasing miR-30c expression (Liu et al., 2018). Moreover, numerous studies have found that SCII was related to a variety of TFs, mRNAs, and signaling pathways which acted significant regulatory effects. In the present study, a comprehensive bioinformatics analysis is applied to construct lncRNA-miRNA-mRNA regulation network and TFs-mRNA regulation network for exploring key lncRNA-miRNA-mRNA axes and TFs in SCII by using GSE138966 from NCBI GEO website and miRNA data from PubMed.

Existing evidence indicated that there are interactions among RNA molecules, such as miRNAs and mRNAs, lncRNAs and mRNAs, and lncRNAs and miRNAs; these RNA molecules collaborate to form a dynamic regulatory network acting as competitive endogenous RNAs (ceRNAs) (Niu et al., 2020). In the present study, a lncRNA-miRNA-mRNA network was built based on the interactions between lncRNA-miRNA, miRNA-mRNA, and lncRNA-mRNA. In the network, 154 lncRNA nodes, 139 mRNA nodes, and 51 miRNA nodes were included. Based on topological analysis of the ceRNA network, 13 key mRNAs, 26 key miRNAs and 30 key lncRNAs were identified. We further found that three upregulated DElncRNAs (NONRATT019236, NONRATT009530.2, NONRATT026999.2) and three downregulated DElncRNAs (TCONS-00032391, NONRATT023112.2, NONRATT021956.2) might be crucial lncRNAs, that controlled the SCII related ceRNA network, from the central network.

In the present study, we found that the upregulated mRNAs were enriched in “negative regulation of MAPK cascade,” “negative regulation of ERK1 and ERK2 cascade,” “p38MAPK cascade,” “stress-activated protein kinase signaling cascade,” and “negative regulation of protein phosphorylation.” KEGG analysis demonstrated that upregulated mRNAs were significantly in “Toll-like receptor signaling pathway,” “MAPK signaling pathway.” It has been proved that SCII promoted activation of MAPK signaling pathways including ERK, p38, JNK (Chen et al., 2008, 2020b; Lu et al., 2010; Fu et al., 2018). Fu et al. (2018) demonstrated that resveratrol protected against SCII by blocking iNOS/p38MAPK signaling pathway. Granulocyte colony-stimulating factor also could reduce the motor function defects following SCII through downregulating phospho-p38 and phospho-c-JNK (Chen et al., 2008). Besides, SCII induced ERK1/2 phosphorylation, followed by neuronal loss through caspase 3-mediated apoptosis and inhibiting ERK1/2 phosphorylation significantly attenuated apoptosis and increased neuronal survival (Lu et al., 2010). Neutralizing TLR4, which was a class of transmembrane proteins that could recognize specific ligands extracellularly, largely reduced blood spinal cord barrier (BSCB) disruption and neuronal apoptosis following SCII (Li et al., 2014). Dexmedetomidine preconditioning stabilized the integrity of BSCB and inhibited the inflammatory response partially by inhibiting the HMGB1-TLR4-NF-κB signaling pathway to protect against SCII (Liu et al., 2019). These studies are in accordance with our bioinformatics analysis.

In addition, we constructed a ceRNA-Pathway network to detect the pathways in which the six crucial lncRNAs and relative miRNAs were involved. In the network, we found that Map3k8, that correlated with NONRATT009530.2 and NONRATT026999.2, involved in multiple signaling pathway such as: “Toll-like receptor signaling pathway,” “TNF signaling pathway,” and “MAPK signaling pathway.” NONRATT026999.2 and NONRATT009530.2 might involve in these key signaling pathways and affected SCII via regulating miR-20b-5p/Map3k8 axis.

Map3k8 has the ability to activate p38α and p38, thus promoting the production of different kinds of inflammatory mediators in neutrophils and might be a potentially anti-inflammatory target (Xu et al., 2018; Li et al., 2020). Map3k8 deficiency reduced neutrophil and macrophage infiltration in the liver of mice treated with acetaminophen (Sanz-Garcia et al., 2013). Map3k8 has also been proved to act as a significant mediator for collaboration of pattern recognition receptors with danger-associated molecular patterns to induce IL-1β and TNF production (Mielke et al., 2009). In acute peripheral inflammation *in vivo*, Map3k8 (−/−) mice demonstrated notably decreased NGF, TNFα, prostaglandin E (2) levels and myeloperoxidase activity (Soria-Castro et al., 2010). A recent study showed that miR-381-3p downregulated Map3k8 to inhibit inflammation and the activation of TNF-α signaling pathway following ischemic stroke (Li et al., 2020). miR-20b-5p has been studied in myocardial I/R injury (Liang et al., 2018; Wang S. et al., 2019; Zhou et al., 2020b). It has been shown that miR-20b-5p promoted ventricular remodeling by targeting the TGF-β/Smad signaling pathway in myocardial I/R injury (Liang et al., 2018). LncRNA MALAT1 promoted oxygen-glucose deprivation and reoxygenation (OGD/R)-induced cardiomyocytes injury through sponging miR-20b-5p to enhance beclin1-mediated autophagy (Wang S. et al., 2019). miR-20b-5p attenuated apoptosis in cardiomyocytes induced by hypoxia via the HIF-1α/NF-κB pathway (Zhou et al., 2020b). miR-20b-5p was also been explored in hepatic I/R injury and knockdown of the expression of HOTAIR attenuated autophagy via the miR-20b-5p/ATG7 axis (Tang et al., 2019). miR-20b-5p has also been studied in inflammatory response. miR-20b-5p inhibited inflammation in mycobacterium tuberculosis through targeting NLRP3/caspase-1/IL-1β pathway (Lou et al., 2017). miR-20b-5p alleviated neuropathic pain and neuroinflammation through the inhibition of Akt3 expression in chronic constriction injury rat model (You et al., 2019). miR-20b-5p reduced inflammatory gene expression in T cells and fibroblasts via working with IL-27 (Figueiredo Neto and Figueiredo, 2017). In the present study, qRT-PCR analysis demonstrated that the expression of NONRATT009530.2, NONRATT026999.2 and Map3k8 were markedly increased in SCII (*p* < 0.05), while the miR-20b-5p expression levels was significantly decreased (*p* < 0.05). Previous studies and our results implied NONRATT026999.2 and NONRATT009530.2 might involve inflammatory response following SCII via miR-20b-5p/Map3k8 axis.

SP1 was suggested to be a widely expressed DNA-binding protein containing a C2H2 zinc finger structure, which modulated gene transcription in diverse physiological and pathological processes (Wang R. et al., 2020). Based on previous literatures, SP1 favored in regulating inflammatory response in diverse diseases (Shin et al., 2015; Yang et al., 2018), and the absence of SP1 attenuated the expression of inflammatory factors IL-6, IL-1β, and TNF-α in hypoxia induced human umbilical vein endothelial cells (Yang et al., 2018). Qin et al. (2018) reported that suppressing the activation of SP1 effectively inhibited OGD/R-induced inflammatory activation in microglial. In addition, SP1 expression was upregulated after acute kidney injury induced by I/R (Chen et al., 2017). SP1 expression was also increased in cortex subjected to brain I/R insult and in primary neurons subjected to OGD/R treatment (Sun et al., 2015). Hnf4a is a TF belonging to the nuclear receptor superfamily and is expressed in the intestine, kidney, liver, and pancreas (including β-cells) (Yamanishi et al., 2015; Sato et al., 2017). It has been proved that Hnf4a was a key TF which showed specific binding at enhancer and super-enhancer sites during ischemic acute kidney injury (Wilflingseder et al., 2020). A study demonstrated that the increase of an acute-phase response from the concomitant action of IL-6, TNF-α, and IL-1β inhibited the expression of Hnf4a -dependent APR genes in HepG2 cells (Wang and Burke, 2008). Besides, in pancreatic β-cells, hypoxia reduced Hnf4a/MODY1 protein expression by activating AMPK (Sato et al., 2017). In the present study, we explored the TFs correlated with the mRNAs of the ceRNA-Pathway network. TFs analysis of ceRNA- Pathway related mRNAs in SCII was constructed. We noted that SP1, Hnf4a, Jun and Olig2 had significant regulatory effects on ceRNA-Pathway related mRNAs. And Map3k8 could be regulated by SP1 and Hnf4a. Moreover, we found that the mRNA expression levels of SP1 and Hnf4a were markedly increased in SCII via qRT-PCR analysis. SP1 and Hnf4a might regulate the inflammatory response in SCII via regulating Map3k8.

## Conclusion

In general, we identified DElncRNAs, DEmiRNAs, and DEmRNAs and selected highlighted lncRNA-miRNA-mRNA axes through a ceRNA network. We also obtained key TFs of ceRNA-related mRNAs. Furthermore, we found that NONRATT026999.2 and NONRATT009530.2 might involve SCII via miR-20b-5p/Map3k8 axis in the complex ceRNA network. As important TFs, SP1, and Hnf4a might regulate Map3k8. However, we realized that this study was implemented by bioinformatics analysis and the results need to be further validated. The mechanisms of NONRATT026999.2 and NONRATT009530.2 regulation of miR-20b-5p/Map3k8 axis are not well established in the current study. Further investigation is necessary to identify how NONRATT026999.2 and NONRATT009530.2 involve SCII via miR-20b-5p/Map3k8 axis. All in all, this study offered a novel insight into the ceRNA network and TFs regulation network in SCII and laid the foundation for further experimental and clinical research.

## Data Availability Statement

The datasets presented in this study can be found in online repositories. The names of the repository/repositories and accession number(s) can be found in the article/Supplementary Material.

## Ethics Statement

The animal study was reviewed and approved by the Ethics Committee of China Medical University.

## Author Contributions

FC and DW designed and performed the experiments and obtained the data. LW, BF, ZZ, and DW performed the statistical analysis and wrote sections of the manuscript. JH wrote the first draft of the manuscript. All authors contributed to manuscript revision, read, and approved the submitted version.

## Conflict of Interest

The authors declare that the research was conducted in the absence of any commercial or financial relationships that could be construed as a potential conflict of interest.

## Footnotes

1 http://www.mirdb.org

2 http://www.microrna.org/

3 http://gtrd.biouml.org

## Supplementary Material

Supplementary Table 1 Differentially expressed lncRNAs in the GSE138966 dataset (*p* < 0.05, | FC| > 1.5).

Supplementary Table 2 Differentially expressed mRNAs in the GSE138966 dataset (*p* < 0.05, | FC| > 1.5).

Supplementary Table 3 The interactions between the DEmiRNAs and DElncRNAs.

Supplementary Table 4 The interactions between DEmiRNAs and DEmRNAs.

Supplementary Table 5 GO and KEGG analysis dataset.

Supplementary Table 6 The information of lncRNAs, mRNAs, and miRNAs in the ceRNA network.

Supplementary Table 7 The information of TFs and predicted mRNAs in TFs-mRNAs regulatory network.
